# Isolated IgG4-related interstitial lung disease: unusual histological and radiological features of a pathologically proven case

**DOI:** 10.1186/2049-6958-8-22

**Published:** 2013-03-19

**Authors:** Thomas Wibmer, Cornelia Kropf-Sanchen, Stefan Rüdiger, Ioanna Blanta, Kathrin M Stoiber, Wolfgang Rottbauer, Christian Schumann

**Affiliations:** 1 Department of Internal Medicine II, University Hospital of Ulm, Albert-Einstein-Allee 23, 89081, Ulm, Germany

**Keywords:** Autoimmune disease, IgG4, Interstitial pneumonia, Lung fibrosis, Usual interstitial pneumonia

## Abstract

IgG4-related lung disease is commonly associated with autoimmune pancreatitis. Recently, isolated IgG4-related interstitial lung disease (ILD) without other organ involvement has newly been reported in two cases with clinical features of nonspecific interstitial pneumonitis (NSIP).

We report the first case of an isolated IgG4-related ILD in a 78-year-old man with dry cough and dyspnea, whose clinical findings proved to be different from NSIP. Serum IgG4 levels were increased. Chest CT scan revealed bilateral consolidations especially in the lower lobes, enlarged mediastinal and hilar lymph nodes and pleural effusions. Video-assisted thoracoscopic (VATS) lung biopsy revealed a pattern similar to usual interstitial pneumonia (UIP) and an abundant IgG4-positive plasma cell infiltration. He was effectively treated by steroid therapy.

Increasing recognition of IgG4 related diseases has led to a growing number of new entities. The novel concept of isolated IgG4-related ILD as a pulmonary manifestation of a systemic IgG4-related disorder should be taken into account as a possible differential diagnosis of ILD and mass-forming lesions, even when no other organ manifestation is clinically apparent at the time of diagnosis. Lung specific diagnostic criteria and algorithms are required to enhance diagnostic accuracy in cases of possible IgG4-related ILD.

## Background

IgG4-related disease (IgG4-RD) is a recently discovered systemic sclerosing disease that is characterized by IgG4-positive plasma cell and T-lymphocyte infiltration of various organs and elevated serum IgG4 concentrations in the majority of patients [1,2]. Tissue fibrosis, obliterative phlebitis and mass-forming lesions due to lymphoplasmacytic infiltrates and sclerosis have been frequently observed [1-3]. Organ involvement typically occurs as autoimmunepancreatitis (AIP), sclerosing cholangitis, sclerosing cholecystitis, sclerosing sialadenitis, retroperitoneal fibrosis, prostatitis, interstitial nephritis and inflammatory pseudotumor (plasma cell granuloma) [1-3]. Several medical disorders previously regarded as distinct organ specific conditions, are increasingly acknowledged as part of a systemic IgG4-related disease [1].

Effective treatment is needed when vital organs are affected because IgG4-RD can lead to serious organ damage [1]. Both the pancreatic and extrapancreatic lesions respond remarkably well to steroid therapy [1,3,4], but recurrence at local and distance sites seems to be common particularly when steroid treatment is tapered [1].

In recent years, IgG4-related interstitial lung disease (ILD) has newly been reported, occurring with and without other organ involvement [4-7].

## Case presentation

### Case report

A 78-year-old man with no significant past medical history presented with a 9 months history of dry cough and shortness of breath upon exertion. He had a short-term history of occupational inhalation of nitro-cellulose in a paint-spray line in 1961, but no history of smoking and no regular drug intake. Physical examination revealed decreased breath sounds and bilateral inspiratory fine crackles in the lower zones. Pulmonary function test demonstrated a restrictive pattern with mildly reduced diffusing capacity (DLCO).

Chest CT showed bilateral, pleural consolidations of the lower and upper lobes with air bronchograms, diffuse ground-glass opacities and traction bronchiectasis, enlarged mediastinal and bilateral hilar lymph nodes, small bilateral pleural effusions and a small pleuropericardial callus on the left side.

Laboratory findings showed increased serum levels of IgG (27 g/l; normal <16 g/l) and IgG4 (3.63 g/l; i.e. 13.4% of total IgG; normal <2.0 g/l and <6% of total IgG). Anti-nuclear antibody level was positive (1:640, homogeneous) and complement components C3c and C4 were decreased (0,63 g/l and <0,02 g/l). Eosinophil count was increased to 0.38 Giga/l (5.6%).

Bronchoalveolar lavage Giemsa stain was not evaluable due to mucus and predominantly destroyed cells.

Pathological examination of a surgical lung biopsy obtained by video-assisted thoracoscopic surgery (VATS) revealed a pattern similar to UIP with chronic lymphoplasmacytic infiltration and significant diffuse interstitial and pleural fibrosis of both reticular, confluent and mass-like aspect, as well as moderate honeycomb changes and significant vascular wall thickening. There was no evidence of vasculitis.

Immunostaining showed infiltration in the interstitium of CD3 positive T-cells and CD20 positive B-cells as well as numerous CD38 positive, IgG4-positive plasma cells. The mean number of IgG4+ plasma cells per high power field (HPF) was more than 10.

Bone marrow aspiration and peripheral blood flow cytometry did not demonstrate a monoclonal lymphoid population nor suggest malignancy.

**Figure 1:**
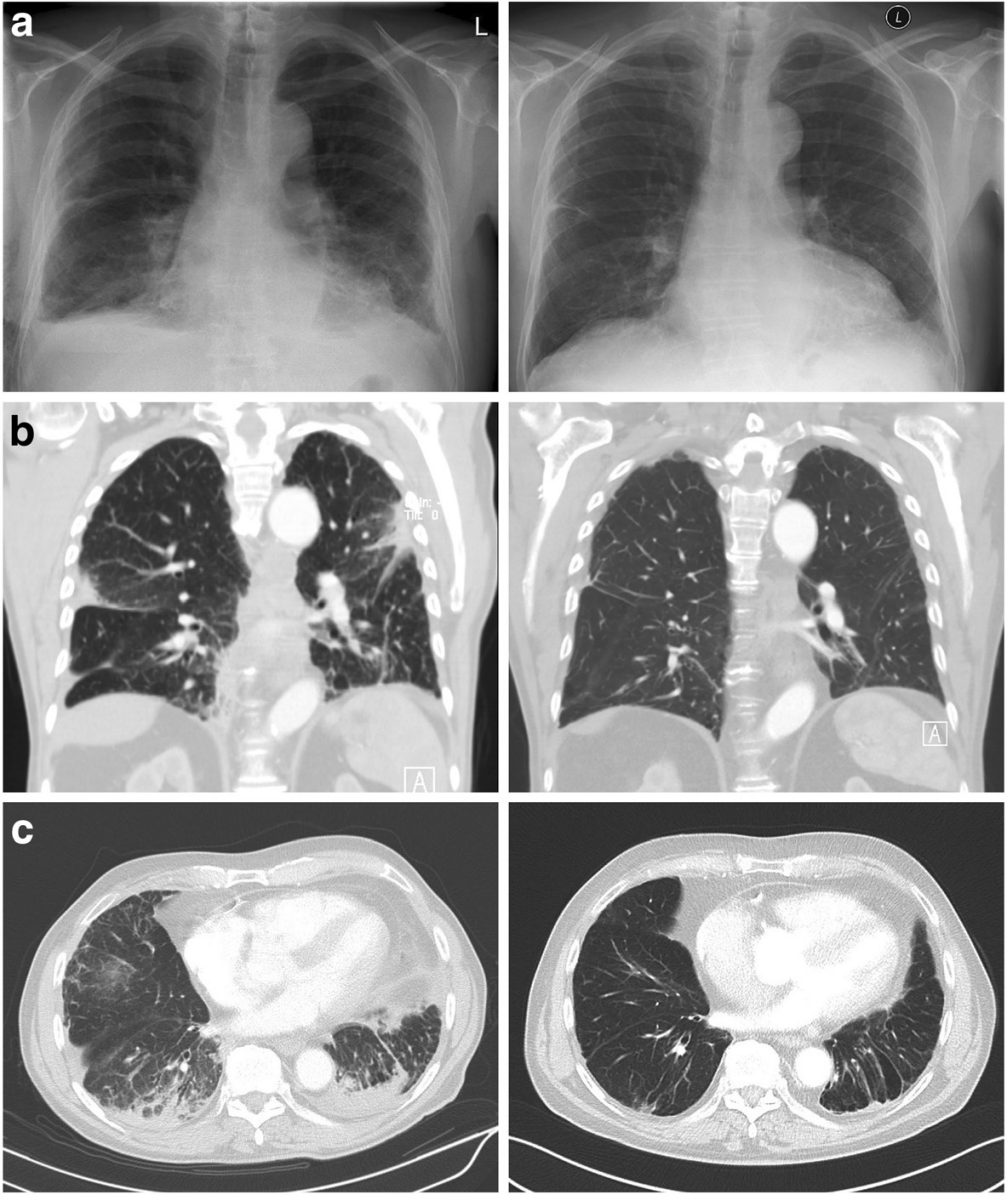
**a: *Left*: Chest radiograph obtained upon admission showed inhomogeneous shadows in the lower lobes, right sided pleural effusion and a pleural consolidation in the right middle field.***Right*: significant improvement after 1 month of steroid therapy. **b** and **c**: *Left*: CT-scan before treatment start showed bilateral pleural consolidations especially of the lower lobes with air bronchograms and traction bronchiectasis. *Right*: significant improvement after 4 months of steroid therapy.

Based on our findings, a final diagnosis of isolated IgG4-related ILD was made. 4 months after initiating a treatment with prednisolone (60 mg/day), the patient showed improvement of symptoms and there was a normalization of IgG4 levels (0.60 g/l; 7.6% of total IgG). Chest x-ray and chest CT (Figure 1) as well as pulmonary function test demonstrated significant improvements.

## Discussion

The first case of ILD associated with IgG4-related disease was reported in 2004 in a 63-year-old man with AIP [8]. In western countries, similar cases of IgG4-related ILD in patients with AIP were reported in 2006 [7].

In 2008, Takato et al. reported the first case of isolated IgG4-related ILD in a 59-year-old man who was found to have ground-glass opacities and reticular shadows with honeycomb-like changes and traction bronchiectasis in the lower lobes on chest CT scan. Pathological examination of a lung biopsy obtained by VATS revealed findings corresponding to fibrotic nonspecific interstitial pneumonitis (NSIP). Subsequent immunostaining for IgG4 showed infiltration with numerous IgG4-positive plasma cells. Serum IgG4 levels were increased. This patient did not present with AIP and the authors suggested that IgG4-related ILD could occur unrelated to AIP [5].

More recently, another case of isolated IgG4-related interstitial pneumonia was reported in a 75-year-old male patient who presented with a two months history of cough and shortness of breath upon exertion. A chest CT scan revealed bilateral diffuse ground-glass opacity with traction bronchiectasis, especially in the bilateral lower lobes. Serum IgG4 levels, which were screened by chance, were increased. Pathologic examination of VATS biopsies revealed infiltration of mostly IgG4 positive plasma cells. There was thickening of the bronchovascular bundles and alveolar septa but neither fibrotic nor vascular lesions were observed. The authors hypothesized that IgG4 plays an important role in the pathogenesis of some cases of interstitial pneumonia initially diagnosed as idiopathic NSIP, and that these interstitial pneumonias may be regarded as a new entity that should be differentiated from idiopathic NSIP [6].

The present case, to our knowledge, is the third case of isolated IgG4-related ILD ever reported and the first observed in western countries. Similar to the cases reported before [5,6], AIP was not clinically apparent in this patient and symptoms as well as radiologic findings and serum IgG4 levels responded well to steroid treatment. Histological pattern was similar to UIP, unlike the two cases published before where NSIP pattern was present. Imaging was characterized by consolidations, lymphadenopathy and pleural effusions, whereas reticular shadows and ground glass opacities were predominant in the previous two cases.

Although a histological finding of UIP often corresponds to a clinical diagnosis of idiopathic pulmonary fibrosis (IPF), it is known that a histological pattern similar to UIP, like in the present case, can also occur in clinical conditions other than IPF [9]. In fact, chest CT findings in this case were strikingly different from the radiologic pattern expected in IPF. Eventually, immunohistology, serum IgG4-concentrations and radiologic features met all diagnostic criteria recently proposed for IgG4-related disease, which led to the definite diagnosis of IgG4-related ILD [3]. Consistently, the rapid and significant response to steroid treatment supported the diagnosis.

Classification criteria and nomenclature of IgG4-RD are currently being established [3]. We doubt that isolated IgG4-related ILD needs to be classified as a new separate entity among idiopathic interstitial pneumonias (IIP), as proposed elsewhere [5,6]. Assuming that IgG4-related ILD may have the same pathogenesis as IgG4-related disease manifesting in other organs, it could be considered as a part of the spectrum of systemic IgG4-related sclerosing diseases, even when no other organ involvement may be clinically apparent at the time of diagnosis. We therefore suggest that IgG4-related ILD may be classified as “diffuse parenchymal lung disease (DPLD) of known cause or association”. Systematic data on the prevalence and incidence of IgG4-related ILD is needed, and further studies addressing the evaluation of IgG4 concentration in tissue and serum in patients with ILD may shed further light on the association of IgG4 with ILD and the underlying pathophysiology.

## Conclusions

Comprehensive diagnostic criteria for IgG4-RD have recently been proposed, including additional organ specific criteria for IgG4+ AIP, renal and Mikulicz’s disease [3]. However, lung-specific criteria and algorithms are required to precisely diagnose cases of IgG4-related ILD with highest possible sensitivity and specificity, since radiologic findings and pathologic specimens may resemble not only NSIP but also those of other interstitial lung diseases and even malignancy [1,3-6].

## Consent

Written informed consent was obtained from the patient for publication of this Case report and any accompanying images. A copy of the written consent is available for review by the Editor-in-Chief of this journal.

## Abbreviations

AIP: Autoimmune pancreatitis; CT: Computed tomography; DLCO: Diffusing capacity of the lung for carbon monoxide; DPLD: Diffuse parenchymal lung disease; HPF: High power field; IgG: Immunoglobulin G; IgG4: Immunoglobulin G subclass 4; ILD: Interstitial lung disease; NSIP: Non-specific interstitial pneumonia; UIP: Usual interstitial pneumonia; VATS: Video-assisted thoracoscopy surgery.

## Competing interests

The authors declare that they have no competing interests.

## Authors’ contributions

TW carried out the literature review, coordination and draft of the manuscript. CK, SR, IB, KS participated in drafting the manuscript and revising it critically. WR and CS have participated in revising the manuscript and have given final approval of the version to be published. All authors read and approved the final manuscript.
